# Preliminary Insights on Barriers to and Facilitators of Healthy Eating for Rural Residents Emerging from Extreme Poverty: A Qualitative Study in Dafang, China

**DOI:** 10.3390/healthcare12131246

**Published:** 2024-06-22

**Authors:** Jian Zhao, Ye Wang, Jing Wu, Qianqian Luo, Bingxia Zhang, Min Zhang

**Affiliations:** School of Population Medicine and Public Health, Chinese Academy of Medical Sciences and Peking Union Medical College, Beijing 100730, China; zhaojian@sph.pumc.edu.cn (J.Z.);

**Keywords:** dietary views, rural residents, barriers, facilitators, dietary transition, socio-ecological model, qualitative research

## Abstract

The purpose of this study was to examine the dietary views and practices and to identify associated barriers to and facilitators of healthy eating of rural residents emerging from poverty in the context of rapid socio-economic development. A qualitative design with semi-structured interviews was utilized to collect detailed insights into the dietary behaviors of 22 rural residents from 10 villages in Fengshan town, Dafang county, Guizhou province, China. Thematic analysis was applied to interpret the interview data, guided by the socio-ecological model. Four themes were identified: traditional eating patterns, factors influencing food choice, limited knowledge of healthy diet, and lack of nutritional guidance. Barriers to healthy eating included traditional but unhealthy foods, limited nutritional knowledge, inadequate understanding of nutritional requirements, overwhelming nutritional information, and limited professional guidance. Facilitators encompassed healthy traditional eating patterns, household composition, home gardening, preference for traditional bean and soy products, improved food supply and availability owing to poverty alleviation efforts, and being open to receiving professional dietary guidance. As a preliminary investigation into the dietary views and practices of this population, the study highlights a significant gap in the nutritional knowledge and guidance available to rural residents in China, emphasizing the need for comprehensive strategies that address the complex socio-ecological factors influencing dietary behaviors.

## 1. Introduction

Nutrition transition, characterized by shifts in dietary patterns and nutrient intake, often parallels the dynamics of urbanization and acculturation, influenced by socio-economic progression [1]. This transition, particularly in developing nations, has rapidly evolved from a “receding famine” pattern (Pattern 3) to one associated with “nutrition-related non-communicable diseases” (NR-NCDs) (Pattern 4). The latter pattern is distinguished by a heightened consumption of total fat, cholesterol, sugars, and refined carbohydrates, compounded by increasingly sedentary lifestyles [2,3]. In the context of China’s rapid economic development, the nation has experienced profound social and economic transformations. Concurrent with these developments, there has been a notable dietary shift among Chinese residents from traditional diets to those characterized by high-fat, high-energy-density, and low-fiber content [4,5]. These changes have led to a marked increase in the prevalence of overweight, obesity, and NR-NCDs, thereby imposing a growing burden of NCDs [4,6].

The disparity in resource allocation, economic development, infrastructure, education, and healthcare between urban and rural areas in China has led to significant concentrations of poverty and malnutrition in rural regions. As of 2012, nearly 100 million people were living below the national poverty line, defined as an annual income of 2300 yuan (approximately 356 US dollars) [7]. In response, China implemented a targeted poverty alleviation strategy, which by the end of 2020 had successfully lifted 98.99 million rural residents out of poverty. This effort led to the removal of 832 impoverished counties and 128,000 poor villages from the poverty list [8]. According to the national survey on poverty alleviation, a significant majority of households in these counties now have access to adequate food year-round, with 98.94% regularly consuming meat, eggs, milk, or soy products [9].

Rural residents who have recently emerged from poverty are potentially susceptible to a rapid nutrition transition, accompanied by lifestyle changes and shifts in health status. Recent studies have highlighted that despite advancements in poverty alleviation, rural communities continue to experience a nutritional transition that may not necessarily align with improved health outcomes [10]. For example, the prevalence of diabetes and hypertension has shown an upward trend. According to the 2020 Report on China’s Nutrition and Chronic Diseases, the prevalence of diabetes increased from 12.3% to 12.6% in urban areas and from 8.4% to 11.1% in rural areas between 2012 and 2018. Similarly, the prevalence of hypertension decreased from 26.8% to 25.7% in urban areas but increased from 23.5% to 29.4% in rural areas during the same period [11]. As modernization and urbanization continue to progress, the burden of NR-NCDs and related health issues is expected to intensify in rural areas.

The link between unhealthy dietary behaviors and chronic diseases is well established, both as a cause and an area of focus for prevention strategy [12,13,14]. Therefore, promoting healthy eating behaviors is recognized as a crucial preventative measure for improving overall health and reducing chronic disease risk factors [15,16]. While some studies have focused on the nutritional and health status of rural residents in China [17,18,19], there is little research specifically targeting populations who have recently emerged from extreme poverty. There remains a significant gap in addressing the needs of this population, especially concerning their increased risk of NR-NCDs [20]. To establish effective policies and interventions that promote healthy dietary practices, a comprehensive understanding of the current dietary behaviors and influencing factors among rural residents is essential.

Moreover, the integration of recent theoretical advancements in behavior change emphasizes the need for multi-level interventions that consider both individual and collective behavioral determinants. The socio-ecological model provides a comprehensive framework for understanding the multifaceted and interactive effects of personal and environmental factors on individual behavior [21,22]. It posits that behavior is influenced by factors operating at multiple levels, including individual (intrapersonal), interpersonal, organizational, community, and policy levels [21]. This model emphasizes the importance of examining the context within which individuals live and make choices, highlighting the role of social influences, physical environments, and policy environments in shaping behaviors. This approach is particularly relevant in the context of rapid socio-economic development and the transition from extreme poverty, as it allows us to explore how changes at the community and policy levels impact residents’ dietary behaviors.

This study aims to examine the dietary views and practices of rural residents, assess their knowledge and understanding of healthy eating, identify the barriers to and facilitators of dietary behavior, and explore potential intervention strategies to support nutrition in this population. Through this research, we hope to provide more scientific evidence for developing targeted strategies to promote healthy eating behaviors of rural residents, thereby expecting to contribute to a decrease in the prevalence of chronic diseases and enhance overall health within this population.

## 2. Materials and Methods

### 2.1. Setting

**Figure 1:**
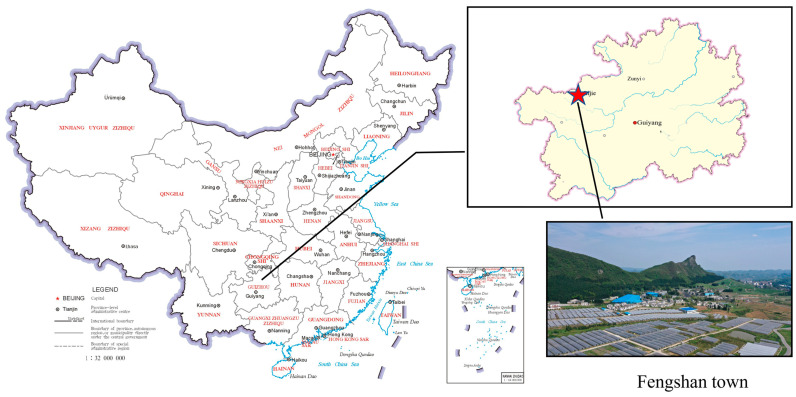
Geographical location of Fengshan town, Dafang county, Guizhou province, China.

This qualitative study was carried out in Fengshan town, Dafang county, a rural area in Guizhou province, China. Guizhou province is among China’s least developed regions, with a significant ethnic minority population. It includes the last nine counties removed from the country’s poverty list, with Dafang county being delisted in late 2019. The county has a population of 857,578, with approximately 62.23% reside in rural areas. In 2020, the per capita disposable income for urban residents was CNY 33,147 (around USD 5206), while that for rural residents was CNY 11,333 (about USD 1780), which is far below the national average. Fengshan town, one of the 16 towns in Dafang county, has a rural population of 13,709, 39.9% of whom belong to ethnic minorities. The average time spent in education in Dafang county and Fengshan town is 7.98 and 7.68 years, respectively [23]. In 2019, there were 9229 diabetes cases and 43,889 hypertension cases in Dafang county, increasing to 11,399 and 49,794, respectively, in 2020 [24,25]. Therefore, the prevalence of non-communicable diseases is low, but the growth rate is rapid. Agriculture is the primary economic activity in Fengshan. Farmers have shifted from subsistence farming to government-aided cultivation of cherry tomatoes, mushrooms, and traditional Chinese medicinal plants (Figure 1).

### 2.2. Design and Participant

This study used a qualitative design with semi-structured interviews conducted in September 2021. The interview questionnaire was developed through an extensive literature review and multiple discussions within the multidisciplinary research team. To ensure the validity and reliability of the interview questionnaire, a pilot test was conducted prior to the main data collection, and necessary modifications were made based on the pilot test results. The final set of open-ended interview questions is available in Supplementary Materials File S1. Local leaders assisted in recruiting of 22 rural residents from all 10 villages in Fengshan. Inclusion criteria were as follows: residents who had not worked outside the town for at least one year; aged 30 years or older, to ensure they had experienced both poverty and poverty alleviation; able to speak Mandarin or a dialect comprehensible to the researchers; and willingness to be interviewed. The study aimed for a diverse sample, considering variations in age, gender, and ethnicity to ensure a comprehensive representation of the community’s demographics.

### 2.3. Data Collection

Face-to-face interviews, each lasting 30–45 min, were conducted at Fengshan’s local community center. Interviewers trained and experienced in qualitative interview techniques, ensured a conducive environment for open discussions. With the consent of the participants, all interviews were audio-recorded, transcribed verbatim, anonymized and stored for confidentiality.

### 2.4. Data Analysis

All digital recordings were transcribed verbatim into mandarin using Iflyrec.com (accessed on 1 March 2022), a professional transcription service. To ensure the accuracy of the transcriptions, researcher B.Z. meticulously cross-verified each transcript against the original audio recordings. The transcriptions were then translated into English by researcher J.W. The translation process incorporated a back-translation method to ensure accuracy, where the English translations were re-translated back into mandarin and crosschecked. Participant confidentiality was safeguarded by anonymizing names with numerical codes in transcripts and quotations.

The study employed Braun and Clarke’s thematic analysis methodology for qualitative data processing [26]. Initial codes were inductively developed by the researcher J.Z. and continually refined during the analysis of each transcript, assisted by NVivo 12.0 software. The coding book is attached as Supplementary Material File S2. Repetitive codes were clustered into main codes, and all codes were then analyzed and broadly categorized into subsequent themes. Each theme was reviewed and refined to ensure it presented a coherent pattern and was substantiated by the coded data. The formulation of themes was closely aligned with the research question, with careful consideration given to their interrelationships and overlaps. Discrepancies in coding and themes were resolved through face-to-face meetings, fostering a consensus-based approach. Data saturation was meticulously assessed and determined when no new themes emerged and ideas became repetitive.

Following the coding and thematic analysis, all sub-themes were classified as barriers or facilitators, determined by their alignment with the scientific discourse on healthy eating and its health implications. Furthermore, these identified barriers and facilitators were systematically categorized into different layers of the socio-ecological system, adhering to a socio-ecological framework as follows: (a) the level of rural residents, (b) microsystem, (c) mesosystem, (d) exosystem, and (e) macrosystem. Discrepancies in interpretations were methodically addressed and resolved through direct face-to-face discussions until a unanimous agreement was achieved.

According to current scholarly discourse on qualitative research methodologies, we implemented several verification strategies integral to our qualitative design to ensure the reliability and validity of our findings [27,28]. Through the use of triangulation, combining interviews with documentary analysis, we cross-validated data points which enhanced the depth and breadth of our insights. Additionally, by incorporating participant validation, we allowed for self-correction in our data interpretation process, making our results more robust and reflective of the participants’ true experiences.

## 3. Results

The study included 22 rural participants from 10 villages in Fengshan town, with a majority being male (63.6%) and of Han ethnicity (59.1%). Most participants had received education up to the 6–9-year level (36.4%) and worked in planting and odd jobs in the village (81.8%) (Table 1).

Four themes were identified through our analysis: traditional eating patterns, factors influencing food choice, limited knowledge of healthy diet, and lack of nutritional guidance (Table 2).

### 3.1. Traditional Eating Patterns

#### 3.1.1. Regular Diet

The participants reported having a regular diet consisting of three meals a day. The meals included wheat or rice-flour noodles for breakfast and dishes with rice for lunch and dinner. Snacks and soft drinks were generally not consumed. A regular diet, no snacks and no soft drinks are considered healthy eating behaviors.

*“In the morning, I usually eat noodles or rice-flour noodles. For lunch, I usually eat rice and two dishes which can be changed with meat or vegetable. Dinner is similar to lunch.”*

(V4)

*“I don’t eat snacks very much. Sometimes I eat some fruit like watermelon and apple. I like to drink tea, no alcohol, and no soft drinks.”*

(V3)

#### 3.1.2. Preference for Local Traditional Food

Almost every participant (21/22) stated their preference for local traditional food, such as “sour bean soup” (kidney beans and sauerkraut), tofu products, and Chinese bacon (dried or smoked pork). However, the health impacts of traditional local foods vary. Sour bean soup and tofu products, which contain a lot of beans and soy, are beneficial to health. On the other hand, Chinese bacon, being a smoked product, is harmful to health. Many participants declared a lack of interest in “Western food” or “new food”, even disdaining food from other parts of China.

*“We like to eat sour bean soup here, and then fry two small dishes for a meal, such as fried dried tofu curd and fried meat slices. We also like tofu pudding. We usually eat them.”*

(V1)

*“Dishes, (we) like shredded potatoes, bacon and bean products in our place.”*

(V11)

*“No, I rarely eat things like hamburgers. I’m not accustomed to the food in other places.”*

(V7)

*“I can accept new fast food like KFC or McDonald’s, but not very often. I don’t particularly want to eat them.”*

(V20)

#### 3.1.3. Eating at Home

Most food was prepared and consumed at home, with Chinese restaurants being chosen for special occasions.

*“We all cook and eat at home and seldom go out to eat.”*

(V5)

*“We eat at home most of the time, and when relatives or friends come, we eat out.”*

(V12)

### 3.2. Factors Influencing Food Choice

#### 3.2.1. Household Composition and Personal Preferences

The participants (15/22) cited household composition was common determinant of food choice, with the person responsible for cooking within the family often deciding what the family ate. Furthermore, personal preferences played a significant role in shaping individuals’ dietary selections. Many participants (13/22) emphasized their tendency to opt for familiar or preferred foods, both for themselves and their families.

*“My parents cook for us. They decide what we eat, but they always ask us what we want to eat. So basically, we eat what we like to.”*

(V3)

*“I choose food by taste of cause, as long as it is delicious.”*

(V1)

*“I decide (what to eat) by myself. I’m used to what I like to eat. Just put more chili.”*

(V16)

#### 3.2.2. Economic Improvement and Increased Family Income

Several participants (8/22) highlighted the substantial impact of economic improvement on food choices, identifying it as the most influential factor. With increased incomes, families no longer faced economic constraints that hindered food availability. Consequently, participants (20/22) reported the ability to afford a wider range of food options that aligned with their preferences.

*“In the past, because the economic conditions were not very good at that time, food variety was unitary. If you fried dishes, you could only choose one single ingredient such as potato or cabbage. Now, because the economic conditions are better, there are three or four ingredients that can be mixed in cooking, and you can eat several dishes in a meal. The main reason for the change is that the economic conditions are better.”*

(V9)

*“I couldn’t eat well seven or eight years ago. I used to eat very little meat at that time. Now it is better. Yes, I think the country’s development is better, so everyone can eat well.”*

(V15)

#### 3.2.3. Food Accessibility

Food accessibility emerged as a notable determinant of food choice among the participants. On one hand, village shops or markets offered a variety of food options. On the other hand, participants reported a preference for consuming vegetables or cereals cultivated on their own farmland or in their own yard. They thought of home gardening as providing them with clean and fresh food.

*“Yes, every village has shops now. And in Fengshan town, there is a large market every week, and we enjoy shopping there.”*

(V12)

*“Sometimes we buy vegetables, meat or some food else from the market.”*

(V8)

*“With the current family conditions, we can eat whatever we want.”*

(V13)

*“We grow vegetables in the countryside where we live. We don’t buy vegetables.”*

(V2)

*“We rarely buy vegetables in rural areas, because we grow them at home.”*

(V15)

### 3.3. Limited Knowledge of Healthy Diet

#### 3.3.1. Limited Knowledge of Healthy Eating

The participants encountered challenges when attempting to provide a comprehensive description of a healthy diet. Out of the participants, a single individual, a mother of two children, explicitly mentioned the importance of incorporating variety, nutrition, and balance in a healthy diet. In contrast, three participants emphasized the significance of increasing fruit and vegetable consumption while reducing meat intake as a characteristic of a healthy diet. Ten participants identified food hygiene and safety as essential components of a healthy diet.

*“I think fruits and vegetables are healthy, and eating meat properly is healthy.”*

(V4)

*“A healthy diet should not be too greasy, and eat more vegetables and fruits.”*

(V20)

*“(Healthy diet means) more hygienic.”*

(V5)

*“I think a healthy diet should check every link of food production. You can’t use pesticides for vegetables planting. Keep them fresh during transportation. Attention should also be paid to artificial breeding.”*

(V8)

*“For example, this kind of food planted by my family without pesticide is relatively healthy. My family has planted a little pepper, melons, cucumbers and tomatoes, which are enough for me to eat, and onions, which are also planted by myself.”*

(V13)

#### 3.3.2. Inadequate Understanding of Nutritional Requirements

The majority of participants (20/22) expressed high satisfaction with their current dietary practices, attributing their contentment to the perception of consuming a nutritious diet that positively impacted their overall health. However, a prevailing theme among the participants was a notable lack of understanding regarding essential nutritional requirements. This observation highlights the participants’ limited knowledge concerning specific dietary needs for optimal health.

*“I feel very good, because I rarely get sick. I feel very satisfied with my current diet.”*

(V9)

*“I think my diet is very good, because there seems to be nothing wrong with my body, you know, people will get sick if they don’t eat well.”*

(V7)

*“I think I eat very well because I have gained several pounds over the past few years.”*

(V10)

### 3.4. Lack of Nutritional Guidance

#### 3.4.1. Overwhelming Information from Friends and Social Media

Approximately half of the participants (13/22) reported receiving intermittent, non-professional advice regarding their dietary choices, primarily from friends or media sources.

*“I have gained weight. So, some friends gave me advices that to eat less dinner and eat seven or eight full.”*

(V13)

*“A few friends of mine sit together and chat. We chat about eating sometimes, they say, it’s better to drink less wine and drink more tea.”*

(V15)

*“I read some information online on my mobile phone.”*

(V3)

*“Yes, for example, sometimes I watch TV program that talked about nutrition, and there are also health experts’ lectures. They talked too much.”*

(V5)

*”The view from socio-media is different, just hard to know who’s right.”*

(V19)

#### 3.4.2. Limited Nutritional Guidance from Primary Care Service

Out of the participants, two individuals disclosed receiving dietary advice directly from doctors. One participant mentioned receiving dietary suggestions based on the results of a physical examination. The second participant reported receiving dietary advice from a doctor during a period of illness. None of the participants mentioned receiving dietary advice from primary care professional or dietitians, despite the presence of at least one general practitioner in each village and a community health service center in the town serving as primary care providers. This observation highlights a significant gap in the utilization of available healthcare resources for dietary guidance.

*“Yes, the doctor’s advice. When I was sick, the doctor advised me to eat less and pay attention to my diet.”*

(V20)

*“I’ve got the physical examination reports with some eating advice from the doctor, for example, he won’t let me eat greasy things. If I eat braised meat, he just let me eat one piece.”*

(V8)

*“No, basically, I don’t go to that place (community health service center).”*

(V8)

*“Very few, because its (community health service center) mind is not on it (nutrition education). They think this is not very important. Its focus is on other aspects.”*

(V18)

#### 3.4.3. Open to Receiving Expert Guidance

Most participants (19/22) were open to nutritional advice, particularly from doctors whom they regarded as reliable professionals. Almost all participants (20/22) were receptive to the idea of dietary advice and willing to consider changes, while acknowledging the difficulties of perseverance.

*“You have to listen to the doctor. What he said is reasonable and based on science. Because somethings said on TV seem a little empty, not real and practical.”*

(V5)

*“If a doctor says so, I’m sure I can accept it and I will change.”*

(V17)

*“If they say what is better for me, I think I can do it. If they say that you are not good at this aspect, you should strengthen nutrition or something, I will also listen to such suggestions.”*

(V9)

### 3.5. Barriers to and Facilitators of Healthy Eating in the Socio-Ecological Model

**Figure 2:**
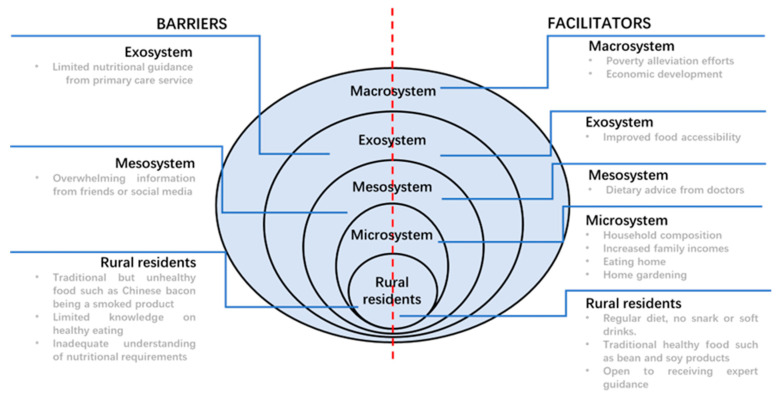
Barriers to and facilitators of healthy eating for rural residents, as adapted from the socio-ecological model.

The socio-ecological model provides a framework within which to conceptualize the barriers and facilitators highlighted by participants, and these are summarized in Figure 2. Our findings reveal that at the level of rural residents, barriers include traditional but unhealthy foods such as Chinese bacon being a smoked product; limited knowledge of healthy eating; and inadequate understanding of nutritional requirements. Conversely, facilitators at this level comprise a regular diet with no snacks or soft drinks, the consumption of traditional healthy foods such as bean and soy products, and a willingness to receive expert guidance. Within the microsystem, facilitators are identified as household composition, increased family income, eating at home, and engaging in home gardening. At the mesosystem level, barriers encompass overwhelming information from friends or social media, while facilitators include dietary advice from medical professionals. Within the exosystem, barriers are observed as the limited nutritional guidance from primary care services, whereas facilitators consist of improved food accessibility. At the macrosystem level, facilitators are identified as efforts toward poverty alleviation and overall economic development.

## 4. Discussion

This study contributes to a better understanding of the dietary views and practices of rural residents who have recently emerged from extreme poverty in the context of rapid economic development. Utilizing the socio-ecological model as a guiding framework, our investigation revealed a range of barriers and facilitators influencing dietary views and practices at multiple levels, from individual knowledge and preferences to broader sociocultural and environmental factors. The findings illustrate the complex interplay of individual, familial, and systemic factors affecting dietary views and practice, highlighting the need for multifaceted strategies to promote healthier eating behaviors.

### 4.1. The Impact of Rapid Economic Development and China’s Poverty Alleviation Efforts on Rural Diets

Food availability is one of the four pillars of food security [29]. In rural China, poverty alleviation efforts have significantly improved the food supply infrastructure, including the construction of transport corridors, modern food markets, and the growth of online shopping [10]. Additionally, accelerated economic development and increasing income levels have improved food accessibility [30]. Consequently, the participants in this study reported being able to afford any food they desired, leading to a perception of dietary satisfaction. However, the impact on dietary quality is more complex. The consumption of high-protein meats, milk, and eggs has substantially increased among rural Chinese residents in recent years [18,31]. Five of the participants in this study also reported eating more meat than ever before, viewing it as a sign of improved quality of life. However, higher incomes often correlate with dietary changes that including higher energy and fat intakes, increased consumption of processed foods and eating out, which can have adverse effects on health [4,32]. Interestingly, the participants in this study rarely ate out (16/22) and often ate no snacks or soft drinks (19/22), preferring to cook fresh food and eat at home. This may be attributed to the relatively low level of urbanization in the area in which our study was conducted. A previous study of dietary differences between sub-urban and rural Chinese villages revealed that shifts to consumption of less staple food and more high-fat red meat was more characteristic of suburban areas than rural villages [33]. This result suggests that as the degree of rural development and urbanization increases, there will be a more rapid transition toward the NR-NCD stage with increased consumption of a high-fat, high-energy-density and low-fiber diets in the absence of powerful measures.

### 4.2. Traditional Food Views and Practices with Both Beneficial and Unhealthy Aspects

The dominance of traditional food views and practices in rural areas, despite the context of rapid socioeconomic change, is highlighted by the present study. Almost every participant stated their preference for local traditional food, such as “sour bean soup”, soy products, and Chinese bacon. Among these traditional foods, beans and soy products are considered beneficial to health, while Chinese bacon, as a smoked processed meat product, is widely recognized as harmful to health. However, there is a notable lack of awareness among rural residents regarding these distinctions. Similar observations have been made in low- and middle-income countries (LMICs) elsewhere, for example, in Colombia [34], the Caribbean [35] and Indonesia [36], where both traditional and modern diets coexist, confirming that dietary transition is a gradual process [37]. As part of traditional practices, the participants in this study also reported cultivating and consuming their own vegetables and grains. Surendran et al. and Auma et al. found that consumption of home-grown food and access to home gardens enabled the consumption of healthier fruit and vegetables with lower environmental impact [38,39]. Therefore, household cultivation in rural areas should be preserved and promoted, which could potentially be an effective strategy for reversing the declining trend of vegetable consumption in rural China.

### 4.3. Inadequate Understanding of the Concept of a Healthy Diet

This study revealed that rural residents have an inadequate understanding of the concept of a healthy diet and their own nutritional requirements. Although there is no universal definition of a healthy diet due to varying nutritional requirements, the consensus is that energy intake should be balanced with expenditure [40,41]. In our study, participants cited food hygiene and safety when asked to describe a healthy diet, with only a single participant mentioning “balance”, indicating a more severe lack of knowledge than previously reported in other of rural Chinese population [42,43]. This study further validates that participants instinctively choose foods based on their own tastes and preferences, underscoring the significant influence of personal preferences in shaping dietary habits, consistent with findings from other studies [44,45,46]. It is well acknowledged that information on health-related behaviors, including diet-related knowledge, can influence individuals’ practices in health management [47,48]. Therefore, without adequate education on dietary nutrition, if people solely rely on personal preferences for food selection, they are inevitably more likely to choose foods that are sweeter or higher in fat content due to their more appealing taste. It is crucial, then, to address nutritional ignorance promptly to promote healthier dietary practices in rural communities.

### 4.4. Lack of Nutritional Guidance

A significant barrier highlighted by the current study is the absence of nutritional guidance, impeding rural residents’ ability to recognize and meet their nutritional needs. The influence of family members on dietary practices was found to be prominent, with participants (12/22) citing friends as an important source of food-related information or advice. Previous studies have also emphasized the influence of social networks, including parents, family and friends, on dietary practices [49,50]. In addition, participants (6/22) indicated new media such as social networking platforms as sources of nutritional and health information. While the internet has become a major source of food and health information, users may lack the critical skills necessary to evaluate nutritional information [51,52]. Furthermore, conflicting and overwhelming information may lead to apathy and a sense of helplessness [53,54]. Participants (19/22) in this study considered clinicians the most reliable source of information, as other sources could not be verified. However, limited clinical advice has been made available, indicating a need for increased access to nutritional guidance. This aligns with previous research in rural China, which identified insufficient support from healthcare professionals and inequitable distribution of public health resources as obstacles to adopting healthy lifestyles [55]. Urbanization has brought many opportunities to rural residents but these have not always been matched by increased health resources. The government should address the paucity of diet related health education in rural areas.

### 4.5. Challenges and Strategies

Improving dietary behavior is a complex and long-term process that requires sustained effort, continuous progress, and iterative improvements. Our research also demonstrates that the influences on dietary views and practices stem from a complex interplay of individual, familial, and systemic factors, indicating that multifaceted strategies are necessary to encourage healthier dietary behaviors. The Chinese government has been proactive in this regard, notably through the comprehensive execution of the “National Nutrition Plan (2017–2030).” In June 2019, the State Council initiated the Healthy China Action Plan, outlining 15 targeted actions, with the “Rational Diet Action” being a key component aiming to foster balanced diets to enhance the quality of life. However, for rural residents emerging from extreme poverty, actions and strategies need to be more targeted. This involves enhancing rural residents’ scientific understanding of local traditional diets and conducting public education for local residents, aiming to preserve elements of the traditional diet that are beneficial to health while discarding or improving those that are harmful. Additionally, at the mesosystem and exosystem levels, improving access to professional nutritional guidance and health resources is crucial. This includes reinforcing healthcare providers’ nutrition training, broadening the reach of nutritional counseling services, and offering personalized dietary advice through mobile health applications. By adopting a comprehensive strategy across these levels, it is possible to effectively promote healthier dietary practices among rural residents, thereby supporting a healthier lifestyle and contributing to a reduction in the rapid increase in nutrition-related non-communicable diseases.

### 4.6. Strengths and Limitations

The current study had several strengths. Firstly, it took place in one of China’s poorest areas, giving valuable insights into the situational context and contributing original evidence to enable the promotion of healthy eating in rural China. Secondly, the timing of the study is pertinent, as the incidence of NCDs in Dafang county remains low but shows rapid growth, indicating the need for early intervention to prevent further increases. Thirdly, we interviewed ethnically diverse rural residents to represent the range of dietary experiences of different minority ethnic populations. However, there are some limitations to our study. The sample size was small, a factor influenced by the geographical setting of Fengshan town, which is located in a mountainous area with a sparse and widely dispersed population across ten villages. This geographic dispersion significantly hindered participant recruitment efforts. Additionally, the study was conducted during the third year of the COVID-19 pandemic. Sporadic outbreaks of COVID-19 severely restricted people’s movement. These conditions further complicated recruitment efforts, leading to a reduced number of participants. Moreover, in rural areas like Fengshan, traditional gender roles frequently impact women’s availability and willingness to engage in activities beyond their immediate familial and community responsibilities, resulting in fewer female participants due to recruitment reluctance. Another limitation is that the study did not include the perspectives of health workers, which could have aided in our understanding of the challenges they face in promoting healthy eating.

## 5. Conclusions

This study investigated the dietary views and practices of rural residents, indicating a significant gap in the nutritional knowledge and guidance available to this population that has recently emerged from poverty, benefiting from poverty alleviation strategies in Dafang, China. Utilizing the socio-ecological model, we systematically categorized the barriers and facilitators affecting the dietary views and practices of rural residents, underscoring the need for targeted and comprehensive strategies to promote healthy eating behaviors. Such strategies include education aiming to enhance nutritional knowledge and personal nutritional requirement at the individual level and improve access to professional nutritional guidance and health resources at the mesosystem and exosystem levels. This research serves as a preliminary investigation into the dietary views and practices of this population. Future studies should investigate these aspects in larger populations and consider additional factors such as the impact of diverse rural environments and incorporate longitudinal designs over time to provide more robust evidence for developing firmer recommendations for this demographic. To conclude, it is essential for Chinese government to take appropriate steps to initiate strategies that improve healthy dietary views and practices, promote a healthier lifestyle, and reduce the rapid increase in NR-NCDs in rural areas of China.

## Figures and Tables

**Table 1:** Characteristics of interviewed rural residents.

Items	Group	N	%
Age (years)	30–39	9	40.9
	40–49	7	31.8
	50–59	6	27.3
Gender	Female	8	36.4
	Male	14	63.6
Ethnicity	Han	13	59.1
	Yi	7	31.8
	Bai	2	9.1
Education (years)	6–9	8	36.4
	9–12	6	27.3
	12–16	7	31.8
	>16	1	4.5
Livelihood	Planting and odd jobs in village	18	81.8
	Local organization staff	4	18.2

**Table 2:** Summary of themes and subthemes.

Themes	Sub Themes
Traditional eating patterns	Routine and traditional dietary pattern
	Preference for local traditional food
	Eating at home
Factors influencing food choice	Household composition and personal preferences
	Economic improvement and increased family income
	Food accessibility
Limited knowledge of healthy diet	Inadequate understanding of nutritional requirements
	Limited knowledge on healthy eating
Lack of nutritional guidance	Overwhelming information from friends and social media
	Limited nutritional guidance from primary care service
	Open to receiving expert guidance

## Data Availability

All data generated or analyzed during this study are included in this published article and its Supplementary Information Files.

## Supplementary Materials

The following supporting information can be downloaded at: https://www.mdpi.com/article/10.3390/healthcare12131246/s1, File S1: Topic guide for interviews with rural residents. File S2: Coding book.
