# Supplementary material for: Preliminary Insights on Barriers to and Facilitators of Healthy Eating for Rural Residents Emerging from Extreme Poverty: A Qualitative Study in Dafang, China
Source: Healthcare (Basel). 2024 Jun 22;12(13):1246. doi: 10.3390/healthcare12131246 (PMC11241384; doi:10.3390/healthcare12131246)
Supplement: Supplementary file 1 [file healthcare-12-01246-s001.zip › healthcare-3034054-Supplementary S2.pdf]

File S2
Coding book

NO.	Code	Description	Example
1	Breakfast items	Foods and beverages consumed for breakfast.	Noodles, rice noodles, and porridge.
2	Lunch items	Foods typically consumed at lunch.	Rice and two dishes of stir-fried vegetables.
3	Dinner items	Foods typically consumed at dinner.	Similar to lunch, occasionally includes a small hot pot.
4	Snack consumption	Types of snacks consumed between meals.	Rarely eats snacks, sometimes eats fruits like apples and seasonal corn.
5	Beverages drunk	Types of beverages consumed and frequency.	Drinks water and tea regularly, occasionally drinks alcohol, typically during evening meals with friends.
6	Primary eating location	Primary locations where meals are consumed.	Eats at home about 50% of the time, occasionally eats at the workplace or with friends.
7	Eating out preferences	Preferences for eating out locations.	Prefers clean, hygienic places; doesn't mind if the taste is not perfect as long as the food is fresh.
8	Factors influencing food choices	Reasons for choosing certain foods.	Chooses food based on freshness rather than price or nutritional content; decisions often made based on family preferences when eating at home.
9	Concept of healthy eating	Personal understanding of what constitutes a healthy diet.	Healthy eating involves food safety and hygiene, prefers homegrown vegetables without pesticides.
10	Self-assessment of dietary health	Personal assessment of their own diet's healthiness.	Considers his diet relatively healthy because it includes fresh, self-produced food.
11	Changes in diet over the years	Description of how dietary habits have changed over the years.	Diet has become more diverse with better economic conditions.
12	Reasons for changes	Why dietary habits have changed.	Economic improvement, increased availability of diverse foods.

13	Trying new foods	Frequency and type of new foods tried.	Occasionally tries new foods like fast food or western cuisine.
14	Weight changes	Changes in body weight over time.	Weight increased by approximately 40 pounds over several years.
15	Health condition changes	Description of any changes in health conditions.	Developed hypertension and diabetes over the past few years.
16	Interaction of illness with diet	How illnesses have influenced dietary choices.	Reduced fatty foods intake due to heart disease.
17	Dietary advice received	Types of dietary advice received and from whom.	Received advice from doctors and nutritionists on reducing sugar intake.
18	Sources of advice	Main sources from which dietary advice was received.	Mostly from healthcare providers and occasionally from online sources.
19	Preferred type of media	Preferred media sources for receiving dietary advice.	Prefers advice from traditional media like television over social media.
20	Community and healthcare worker advice	Trust in advice given by community health workers and doctors.	Highly values advice from local community health events.
21	Acceptance of advice	Willingness to accept and implement dietary advice.	Accepts and tries to follow dietary advice especially if it aligns with health needs.
22	Help needed for change	Types of support needed to make dietary changes.	Needs guidance and ongoing support from a nutritionist.
23	Longevity of changes	Sustainability of dietary changes made.	Struggles to maintain dietary changes without ongoing support.
