# Supplementary material for: Preliminary Insights on Barriers to and Facilitators of Healthy Eating for Rural Residents Emerging from Extreme Poverty: A Qualitative Study in Dafang, China
Source: Healthcare (Basel). 2024 Jun 22;12(13):1246. doi: 10.3390/healthcare12131246 (PMC11241384; doi:10.3390/healthcare12131246)
Supplement: Supplementary file 1 [file healthcare-12-01246-s001.zip › healthcare-3034054-supplementary.pdf]

File S1:

Topic guide for interviews with rural residents

- Explain why we are doing the research
- Read and understood information sheet. Opportunity to ask questions
- Audio recording
- Confidentiality and exceptions to this
- Information about types of questions will be asking, no right or wrong answers
- Consent form

1．Can you tell me what you eat in a typical day?

2. Where do you tend to eat your meals?
3. How do you decide what kind of food to eat?
4. People often talk about having a ‘healthy diet’. What does that mean to you?
5. Are there any changes of your diet in recent years? If yes, what and why?
6. Are there any changes of your weight and health condition in recent years?
7. Have you ever received dietary advice on what to eat and how to eat?
8. Which sources of dietary advice would you prefer to listen to?
9. If a Dr or community health worker or nutritionist or dietitian recommended that you should eat more health, what would you think of that?
